# Homeosis in a scorpion supports a telopodal origin of pectines and components of the book lungs

**DOI:** 10.1186/s12862-018-1188-z

**Published:** 2018-05-21

**Authors:** Zhiyong Di, Gregory D. Edgecombe, Prashant P. Sharma

**Affiliations:** 1 Key Laboratory of Invertebrate Systematics and Application, College of Life Sciences, Hebei University, Baoding, 071002 Hebei China; 2 Department of Earth Sciences, The Natural History Museum, Cromwell Road, London, SW7 5BD UK; 3 Department of Integrative Biology, University of Wisconsin-Madison, 352 Birge Hall, 430 Lincoln Drive, Madison, WI 53706 USA

**Keywords:** Abdominal appendages, Ancestral gill, Arthropoda, Book lungs, Homeosis

## Abstract

**Background:**

The morphological and functional evolution of appendages has played a key role in the diversification of arthropods. While the ancestral arthropod appendage is held to be polyramous, terrestriality is associated with the reduction or loss of appendage rami, which may obscure the homology of different appendage derivatives. Proxies for appendage homology have included surveys of cross-reactive antibodies for wing markers like Nubbin/PDM, which have suggested that the abdominal appendages of arachnids (e.g., book lungs, tracheal tubules) are derived from ancestral gills (epipods).

**Results:**

Here, we discovered a rare case of inferred homeosis in a scorpion in which the bilobed genital opercula and the pectines are transformed to walking legs, and an abnormal sternite shows a book lung close to an everted structure comparable to the morphology of some Palaeozoic scorpion fossils.

**Conclusions:**

The observed morphology is consistent with abnormal expression of homeotic genes during embryonic development. The phenotype of this abnormal specimen suggests that the genital opercula, the pectines, and parts of the book lung may be derived from the telopodite of abdominal appendages rather than from epipods. This interpretation contradicts the “ancestral gill” hypothesis but reconciles features of the Palaeozoic scorpion fossil record with the embryology of modern scorpions.

## Background

The “ancestral gill” hypothesis proposes that fundamentally different arthropod trunk organs, including chelicerate respiratory organs, evolved from the same ancestral structure, epipodal gills, in parallel instances of terrestrialization [1]. Within chelicerates, morphological investigations of scorpion and horseshoe crab embryogenesis and respiratory organ ultrastructure have supported the idea that arachnid book lungs are derived from internalized book gills, and book lungs in turn may constitute a stepping stone in the evolution of tubular tracheae in derived spiders and apulmonate arachnids (e.g., harvestmen, ticks) [2, 3]. However, the ancestral gill hypothesis is inconsistent with the arthropod fossil record, in that epipods (the outermost ramus of the polyramous arthropod appendage) have been reconstructed as derived, rather than as ancestral features of arthropods [4]. Given the scarcity of developmental genetic data in non-spider chelicerates, little is known about the book gill/book lung transition beyond morphological comparisons.

## Results

### Normal morphology and structures of extant scorpions

**Fig. 1:**
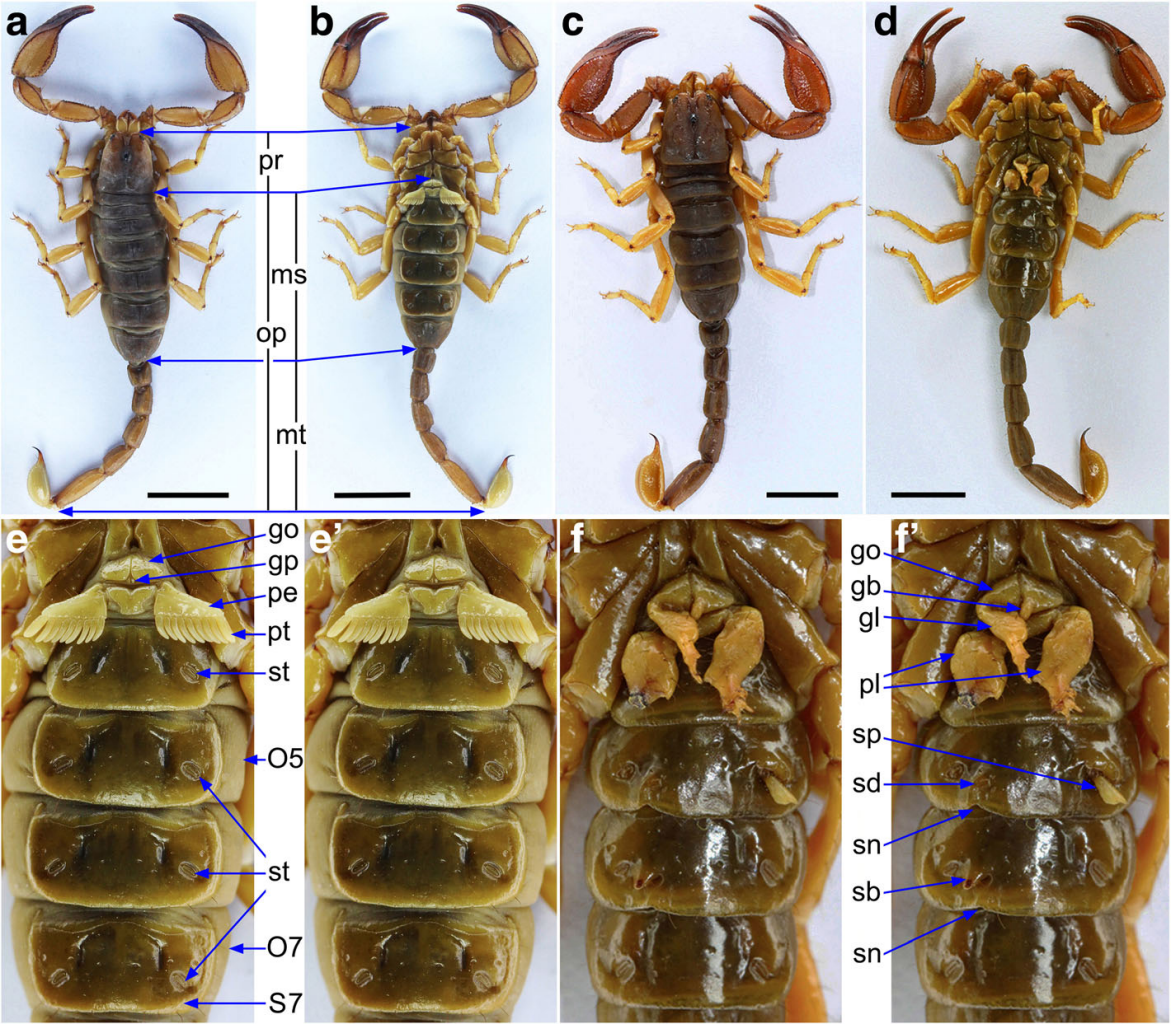
Comparison of wild type individual (**a**, **b**, **e**, **e’**) and inferred homeotic scorpion (**c**, **d**, **f**, **f’**), both immature males of *Scorpiops luridus* from the same locality. (**a**, **b**) Dorsal and ventral views of wild type male showing mesosoma (ms), metasoma (mt, the posterior opisthosoma), opisthosoma (op) and prosoma (pr)*.* (**c**, **d**) Dorsal and ventral views of the homeotic male. The dorsal aspects of the two specimens are similar, but the ventral mesosomae differ. (**e**) Ventral view of normal male, showing genital operculum (go), genital papillae (gp), O5 & O7 (opisthosoma 5 and opisthosoma 7), pectinal lamellae (pe), pectinal teeth (pt), sternite 7 (S7), and stigmata (st). (**e’**) Same as **e** but unlabeled. (**f**, **f**’) Unlabeled (**f**) and labeled (**f’**) ventral views of the homeotic male showing the everted structure (sp, sternal protrusion) and its partially developed bud (sb), genital operculum leg (gl) and its partially developed bud (gb), pectinal leg (pl), sternal depression (sd), and sternal notch (sn). Scale bars: a–d, 10 mm

**Fig. 2:**
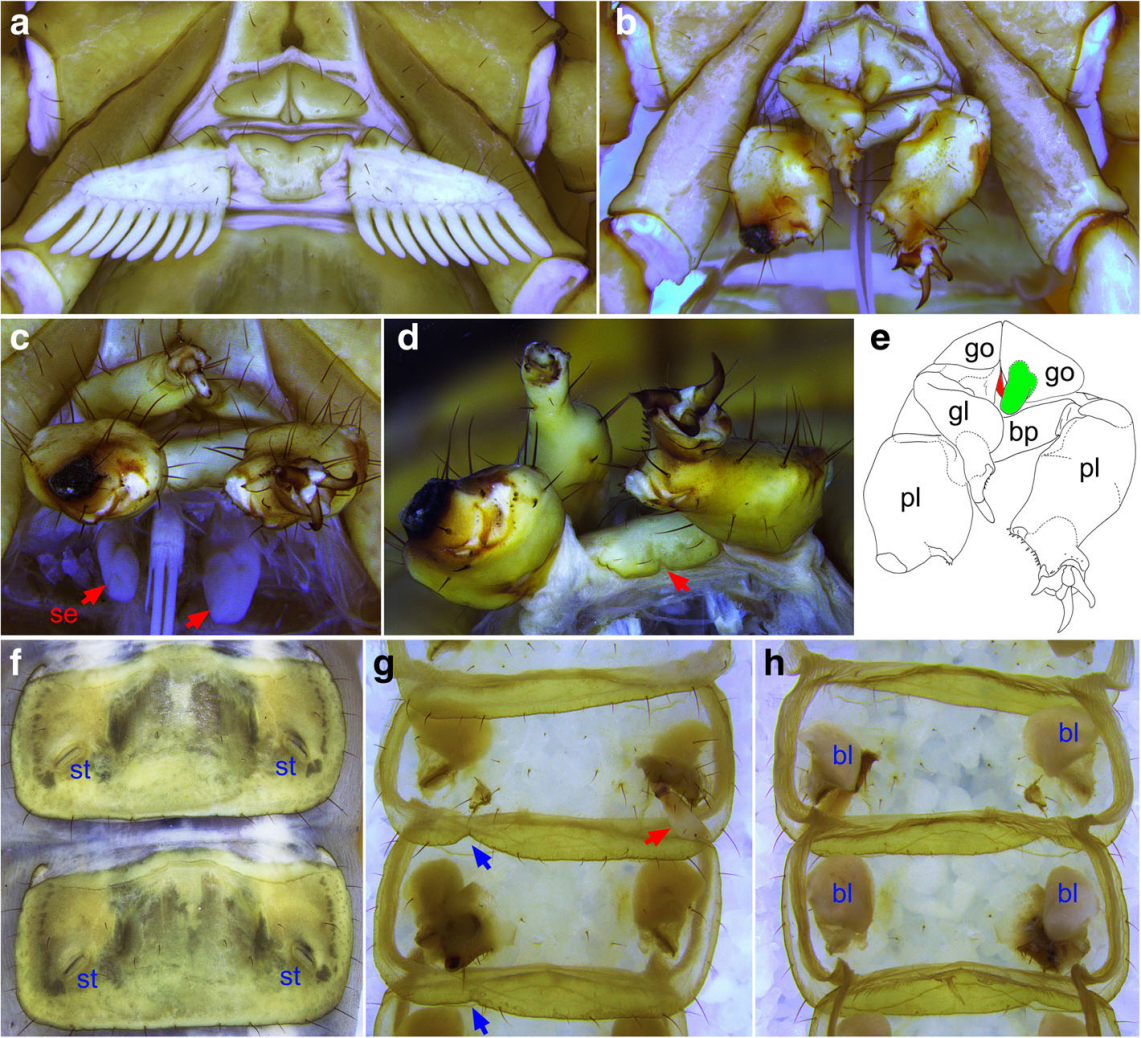
Wild type (**a**, **f**) and inferred homeotically transformed (**b**–**e**, **g**, **h**) structures of the mesosoma. (**a**) The wild type genital operculum is bilobed and anterior of the wing-like pectines. (**b**) In this specimen, the right genital operculum (go) bears a partial walking leg (gl) with telotarsal macrosetae on the distal edge (see **e** for labels). The left genital operculum bears a partially developed bud (green structure). Both pectines are transformed into legs (pl), with clear segmentation and a pair of tarsal claws on the left pectine. (**c**) Two developing spermatophores (se, red arrows) of the homeotic mutant have different sizes. (**d**) A triangular notch (bn) on basal piece of the homeotic scorpion. (**e**) Drawing of B showing homeotically transformed structures, red structure is genital papilla; bp, basal piece. (**f**) Sternite 5 and sternite 6 of a wild type individual showing symmetrical margins and stigmata (st). (**g**, **h**) External (**g**) and internal (**h**) views of homeotic sternite 5 and sternite 6, showing book lung (bl), everted structure (red arrow) and notches (blue arrows)

**Fig. 3:**
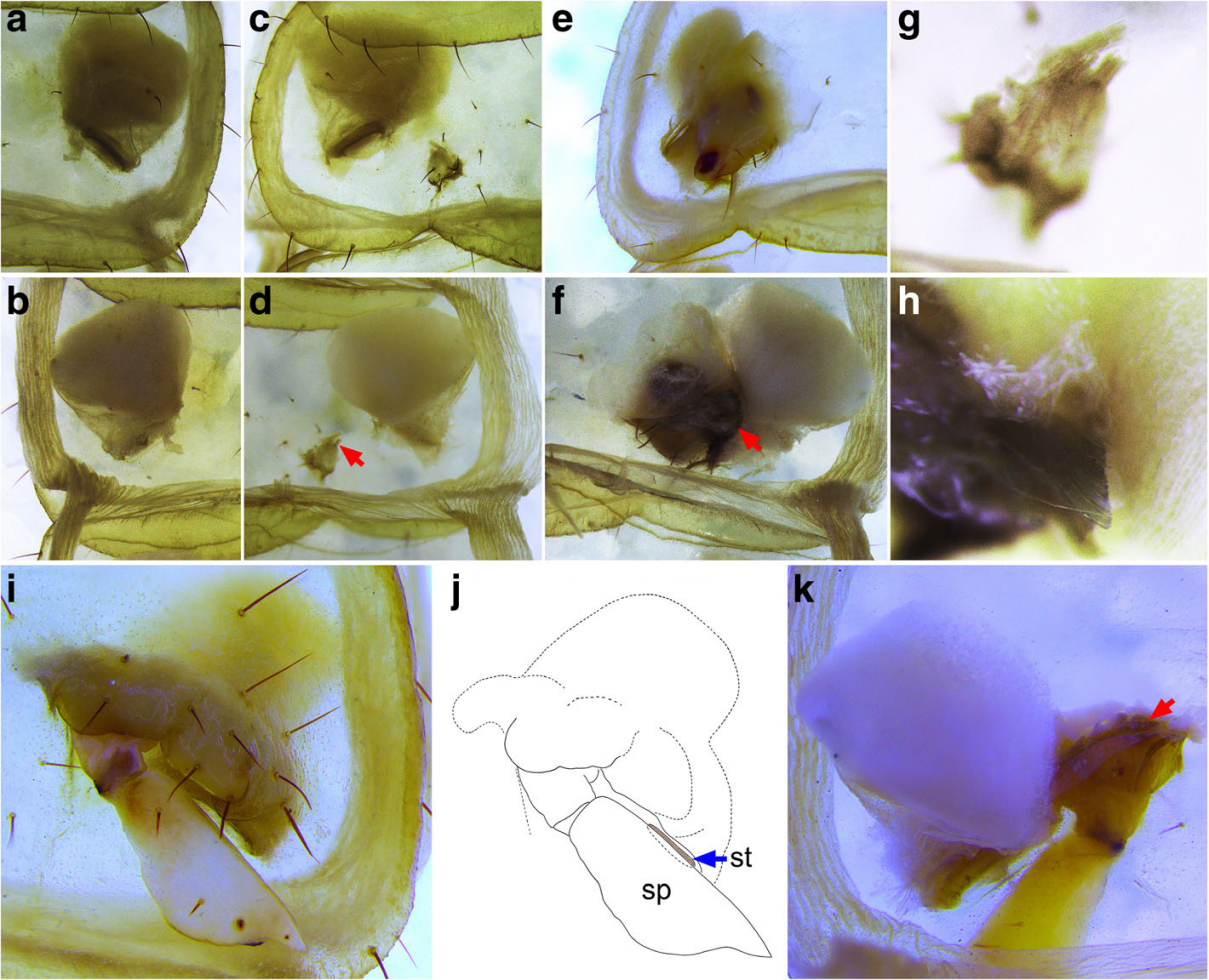
Wild type (**a**, **b**) and inferred homeotic mutant (**c**–**k**) book lung segments. (**a**, **b**) External (**a**) and internal (**b**) views of wild type stigmata on left area of sternite 6. (**c**, **d**) External (**c**) and internal (**d**) views of homeotic stigmata on right of sternite 5. (**e**, **f**) External (**e**) and internal (**f**) views of homeotic stigmata on right of sternite 6. (**g**, **h**) Myofibers developed at the internal roots of O5 depression (**g**, red arrow in **d**) and O6 protrusion (**h**, red arrow in **f**). (**i**–**k**) External (**i**, **j**) and internal (**k**) views of homeotic stigmata on left of sternite 5, showing myofibers (red arrows) and stigmata (st, blue arrow)

The scorpion specimen reported here is an immature male belonging to the species *Scorpiops luridus* Qi et al., 2005 (Scorpiones: Euscorpiidae: Scorpiopinae) (Fig. 1a–f'), from Nêdong County, Xizang, China. In the embryos of extant scorpions, the anterior opisthosoma (the “abdomen” of arachnids, termed the mesosoma (ms) in scorpions) consists of eight segments (O1–O8) [5]. O1 gradually disappears; bilaterally symmetrical anlage fuse to form the genital operculum (go) on O2; pectine lamellae (pe) and teeth (pt) are present on O3 in normal wild type individuals (Figs. 1e, e' and 2a). Pairs of book lungs (bl) and their stigmata (st) are formed on O4–O7, and O8 tapers posteriorly to join with the segmented tail, the posterior opisthosoma (metasoma, mt; Figs. 1e, e' and 3a, b).

### Genital opercula of scorpions initially developed from the proximal part of the genital appendages in their ancestors

**Fig. 4:**
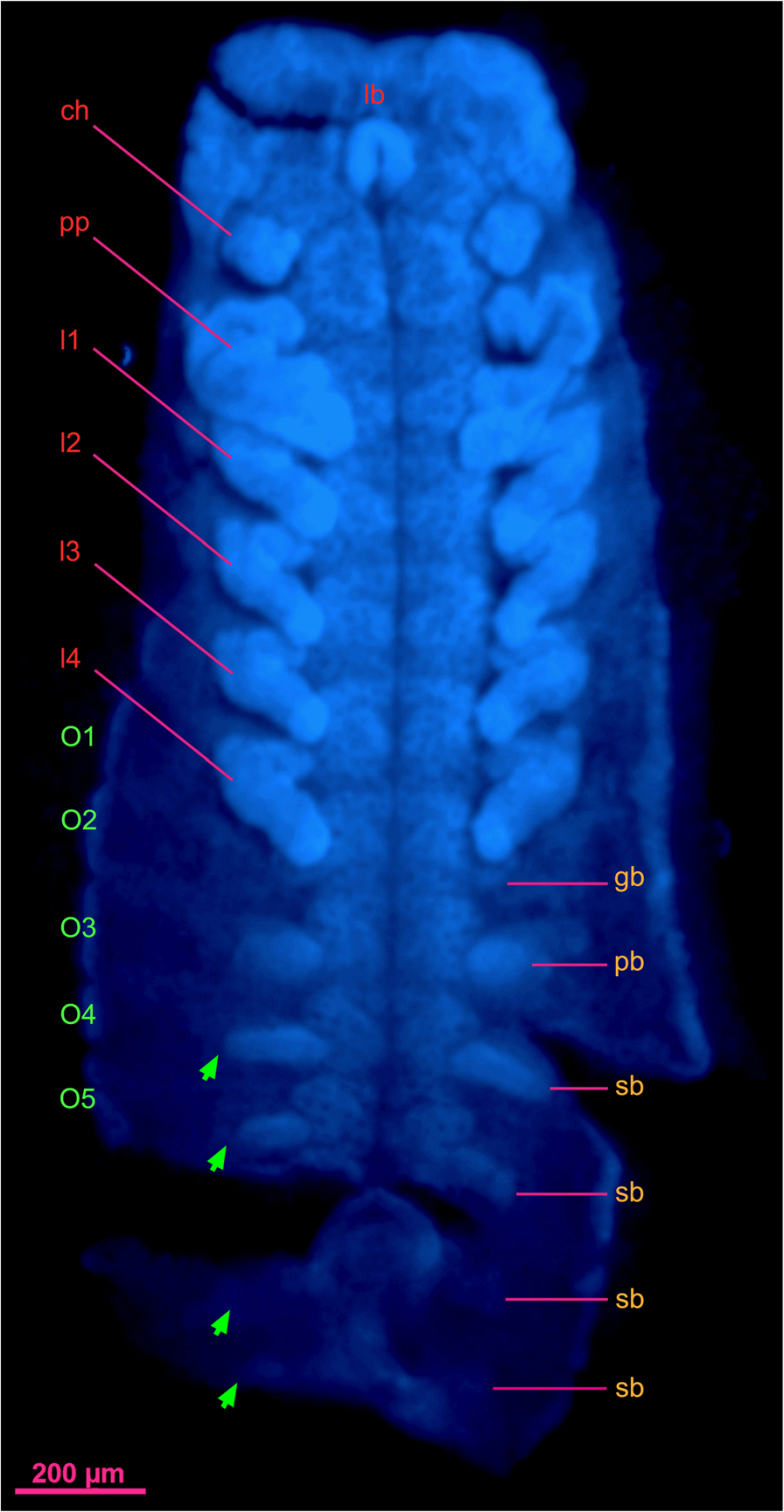
Embryonic stage of *Centruroides sculpturatus* (Arizona bark scorpion) with limb buds. ch, chelicera; gb, partially developed bud of genital operculum leg; lb., labrum; l1–l4, leg 1–leg 4; O1–O5, opisthosoma 1–opisthosoma 5; pb, partially developed bud of pectine; pp., pedipalp; sb, partially developed bud of sternal protrusion. Green arrows show the position where stigmata will develop. Scale bar is 200 μm

The genital operculum of wild type males forms from limb bud-like anlage [6] (Fig. 4), and the longitudinal midline suture is retained in this species (Fig. 2a). In a specimen of *S. luridus* that we interpret as homeotic, the genital opercula are asymmetrical (Fig. 2b). The right genital operculum bears a partially formed leg (gl, genital operculum leg), with typical telotarsal macrosetae, but no terminal claws. The left genital operculum is an incrassate papilla, suggesting a partially developed limb bud (gb), but the proximal connection to the operculum occurs medially, not laterally (Figs. 1f, f' and 2b, e). The two genital papillae (gp) resemble those of wild type conspecifics. In various chelicerate groups, O2 has a reproductive function and bears genital appendages [7]. Dissection of the inferred homeotic mutant revealed two small lobed structures, identified as developing spermatophores (se) (Fig. 2c). The left lobe is somewhat larger and more posterior than the right one (Fig. 2c). The inferred genital operculum-to-leg transformation is consistent with genital opercula of scorpions initially developing from the proximal part of the genital appendages on their ancestors. In particular, this is inferred to have been the coxa, as inferred from the retention of an intact operculum at the base of the specimen’s O2 appendage (i.e., the region corresponding to the coxa).

### The pectines of scorpions are homologous with telopodites

Instead of the distinctive pectines on O3 (IX segment) of normal extant scorpions (Figs. 1e, e' and 2a), the inferred homeotic mutant bears a pair of asymmetrical, partially formed legs (pl, pectinal leg; Figs. 1f, f' and 2b–e). The right pectine has a large, lobe-like structure that bears a setal pattern characteristic of walking legs, and a small number of macrosetae typical of wild type telotarsi on the distal part of the inner surface. The left pectine has external segmentation at the base of the appendage, as well as a pair of well-developed tarsal claws, and a row of macrosetae on the ventral surface characteristic of wild type walking legs. In contrast to wild type counterparts (Fig. 2a), the basal piece (bp) of the homeotic scorpion is setose and has a triangular notch (bn) at the posterior edge (Figs. 1f, f and 2b–e). The homeotic pectine-to-leg transformations suggest that much or the entirety of this unique organ of scorpions is homologous to a telopodite.

### Book lungs of scorpions include components of telopodites

The sternites (S) of normal O5 and O6 have smooth posterior margins (Figs. 1e, e' and 2f). In contrast, the same regions of the inferred homeotic specimen have depressions (sd, sternal depressions) and notches (sn, sternal notches) (Figs. 1f, f', 2g, h, 3c–f). Comparing the region around the book lung stigmata to wild type counterparts (Fig. 3a, b), the left side of O5 sternite and right side of O6 sternite of the homeotic scorpion have an everted structure (sp, sternal protrusion, hypogenetic appendage) and a partially developed bud (sb, sternal bud, hypogenetic appendage; Figs. 1f, f', 2g, h, 3e, i, j). Together with the embryological origin of the book lung as an invagination in the posterior margin of an opisthosomal limb bud [8], the everted structure in this specimen suggests that some component of the book lung represents a telopodite derivative. The left O5 stigma and right O6 stigma are located in sockets in the vicinity of the sternal protrusion (Figs. 2g, h and 3e, f, i–k). Myoanatomy of the mesosoma includes myofibers developed at the roots of the O5 depression and everted structure, and of the O6 protrusion (Fig. 3g, h, k). Book lungs of extant scorpions have a comparable appendicular origin as those of spiders and the book gills of horseshoe crabs [2, 8, 9], the latter having a clearly defined, segmented telopodite (the xiphosuran embryo’s “median lobe”, sensu Farley [9]; Fig. 4b in [10]). In other chelicerates, six paired gills occur on O2–O7 of the Silurian *Dibasterium*†, four paired gills on O4–O7 of Xiphosurida and Eurypterida†, two pairs of book lungs on O2 and O3 of Trigonotarbida†, Araneae, Amblypygi, and Thelyphonida, and a single pair of book lungs on O2 of Schizomida [7]. This distribution of book lungs in Arachnida has been interpreted to mean a single terrestrialisation event of book lungs in its stem lineage [11, 12], though phylogenomic studies alternatively favor a single derived origin of book lungs [13].

## Discussion

Teratological malformations in scorpions, especially developmental anomalies, have been documented in various studies [14, 15]. These can be divided into three types: I, deformation or ontogenetic defects; II, duplication; and III, gynandromorphy and hermaphroditism. Empirical examples of type I teratologies span defects of the pedipalp, including a lack of keels and trichobothria, or a malformed finger; various hypogenetic walking legs and pectines, with deformed, fused, shortened, or missing podomeres and/or pectine teeth [14–18]. More common is the duplication of various body parts (type II), such as two telsons, duplication of the metasoma, and complete distal duplication of the mesosoma, whereas two aculea and duplication of the anterior part of the body is less common [14, 15, 19]. Type III is comparatively rare, examples including both embryos and hemispermatophores, or in the pectines in both sexes [14, 15, 20]. Two similarities are noted among the three types of scorpion developmental anomalies described heretofore: no new structures appear (i.e., de-repression), and the abnormal structures occur in the same locations as wild type counterparts. Thus these developmental anomalies differ from homeosis, the transformation of one organ into another, which arises from misexpression of homeotic genes, which specify the development of organs along the anteroposterior body axis [21].

In tandem with homeotic transformation phenotypes, additional insights into scorpion appendicular homology are provided by examination of the chelicerate fossil record. A similar feature to the triangular notch at the posterior edge of the basal piece of the inferred homeotic scorpion is observed in the first visible sternite of *Eramoscorpius brucensis*†, one of the oldest known fossil scorpions [22]. In other Palaeozoic scorpions, abdominal plates (in bilobosternous scorpions) [23, 24], gill-like structures (in *Waeringoscorpio*†) [25], and bulges (in *Palaeoscorpius devonicus*†) [26] are potential homologs of the everted structures (sternal protrusions) of the homeotic *Scorpiops luridus*. Each sternite of the Palaeozoic scorpion grade “Branchioscorpionina” has two invaginations that were previously interpreted as gill slits [23, 24]. Similar structures also occur in the early embryonic development of extant scorpions [8, 27].

The common ancestor of crown group arthropods is widely reconstructed as bearing a homonomous trunk with paired appendages on each segment [28]. Paralleling this hypothesis, during embryonic development of extant scorpions, opisthosomal organs first take the form of limb buds on the embryonic ventral mesosoma (Fig. 4), prior to differentiation and morphogenesis of pectines and book lungs [5, 6, 27, 29]. The condition of the inferred homeotic scorpion is consistent with the de-repression of posterior limbs. One well-characterized putative mechanism for this transformation is disruption of posterior Hox gene signaling. Specifically, loss-of-function phenotypes of *Antennapedia* and *Ultrabithorax* in a spider result in ectopic appendages on the first opisthosomal segment, and small outgrowths on the book lung segment [30]. Comparable de-repression of abdominal limbs can be incurred in insects upon knockdown of *Ultrabithorax* and *abdominal-A* [31]. Such experimental tractability is not possible in scorpions due to peculiarities of their natural history, such as live birth and long gestation periods [6]. For this reason, the inferred homeotic specimen described here provides clues as to the developmental origins of the enigmatic, gill-like pectine. The morphological transformations between wild-type and inferred homeotic phenotypes suggest that the scorpion genital operculum and pectine are serially homologous to walking legs (telopodites). This is consistent with these structures’ development in the ventral and posterior component of segments marked by *engrailed* expression, as is likewise the case for telopodites [32]. We submit that at least part of the book lung (namely, the operculum) may be serially homologous to telopodites as well, a condition that is comparable to the small inner telopodite of the gills and gill operculum of modern horseshoe crabs [4, 10]. This is consistent with gene expression data that support positional homology of respiratory structures of arachnids in general (book lungs and tubular tracheae) with telopodites [32]. The placement and identity of posterior appendages in Paleozoic chelicerates exhibit considerable diversity [7]. Our data suggest that all these are linked in a single transformational series as telopodites.

## Conclusions

A case of inferred homeosis in a scorpion involves transformations of appendicular structures on two body segments - the bilobed genital opercula and the pectines - to walking legs, and an abnormal sternite bears an everted appendicular structure associated with the book lung. The phenotype of this anomalous specimen is consistent with the inference that the genital opercula, pectines, and at least some parts of the book lung are serially homologous to walking legs of abdominal appendages (telopodites), rather than from epipods, as was favoured by the “ancestral gill” hypothesis. The signal from the inferred homeotic scorpion is consistent with recent gene expression data for scorpions and other arachnids that suggest diverse structures in the posterior part of the arachnid body are linked in a single transformation series, as modified walking legs.

## Methods

Specimens were fixed in 75% alcohol. Photographs were taken with a Canon 650D camera and a Leica M205FA stereomicroscope fitted with a Leica DFC495 digital camera. The staining reagent for scorpion embryos is Hoechst 33,342.

## Abbreviations

bl: Book lung
bn: Notch
bp: Basal piece
ch: Chelicera
gb: Genital operculum limb bud
gl: Genital operculum leg
go: Genital operculum
gp: Genital papillae
l: Leg (l1–l4, leg 1–leg 4)
lb: Labrum
ms: Mesosoma
mt: Metasoma
O: Opisthosoma (O1–O8, opisthosoma 1–opisthosoma 8)
op: Opisthosoma
pb: Partially developed bud of pectine
pe: Pectine lamellae
pl: Pectinal leg
pp: Pedipalp
pr: Prosoma
pt: Pectine teeth
S: Sternite (S7, sternite 7)
sb: Partially developed bud of everted structure (sternal protrusion)
sd: Sternal depression
se: Spermatophore
sn: Sternal notch
sp: Everted structure, sternal protrusion
st: Stigmata
